# Supplementary material for: The Effects of Six-Weeks Change of Direction Speed and Technique Modification Training on Cutting Performance and Movement Quality in Male Youth Soccer Players
Source: Sports (Basel). 2019 Sep 6;7(9):205. doi: 10.3390/sports7090205 (PMC6783855; doi:10.3390/sports7090205)
Supplement: Supplementary file 1 [file sports-07-00205-s001.pdf]

sports

MDPI

Supplement information

The Effects of Six-Weeks Change of Direction Speed and Technique Modification Training on Cutting Performance and Movement Quality in Male Youth Soccer Players

Thomas Dos'Santos *, Alistair McBurnie, Paul Comfort and Paul A. Jones

Directorate of Sport, Exercise & Physiotherapy, University of Salford, Salford, Greater Manchester M6 6PU, UK; a.McBurnie@edu.salford.ac.uk (A.M.); p.comfort@salford.ac.uk (P.C.); P.A.Jones@salford.ac.uk (P.A.J.)

* Correspondence: t.dossantos@edu.salford.ac.uk; Tel.: +44-161-295-2371

Table S1. 6-week COD speed and technique modification training programme.

		Session 1				Session 2			
**Week**	**COD Emphasis**	**Drills**	**Intensity (perceived speed)**	**Total Distance (m)**	**Number of Decelerations and CODs**	**Drills**	**Intensity (perceived speed)**	**Total Distance (m)**	**Number of Decelerations and CODs**
**Week 1**	• Drills specific to deceleration phase, before adding turn and reacceleration	1. 10 × 5-m acceleration to deceleration (2 reps a go)<br>2. 12 × 5-m acceleration to side-steps (20-60°) – 5-m exit to deceleration – 2 reps at 20°, 40°, 60°<br>3. 8 × 5-m acceleration to 135° turns – 5-m exit to deceleration	50-75%	250	25 and 20	1. 10 × 5 m acceleration to deceleration (2 reps a go)<br>2. 12 × 5 m acceleration to side-steps (20-60°) – 5-m exit to deceleration – 2 reps at 20°, 40°, 60°<br>3. 8 × 5-m acceleration to 135° turns – 5-m exit to deceleration	50-75%	250	25 and 20
**Week 2**	• Submaximal/ pre-planned emphasising key aspects of technique<br>• Progressive increase in COD angle and approach velocity	1. 8 × 5-m acceleration to deceleration<br>2. 14 × 5-m acceleration to side steps (45-90°) – 5-m exit to deceleration<br>3. 8 × 5-m acceleration to 135-180° turns – 5-m exit to deceleration	75%+	230	30 and 22	1. 4 × 5-m acceleration to deceleration<br>2. 4 × 10-m acceleration to deceleration<br>3. 3 × 80° zig-zag runs – (2 cuts a rep)- 5-m between cuts to 5-m deceleration (60% effort)<br>4. 4 × 80° zig-zag runs – (2 cuts a rep) – 5-m between cuts to 5-m deceleration<br>5. 2 × 80° zig-zag runs with 180° turn– 4 cuts per trial – 5-m between cuts	100%	285	17 and 30

						6. 3 × 5-m approach to 180°, 5-m approach to 180°, to 10-m deceleration			
**Week 3**	• Pre-planned drill performed maximally<br>• Introduction of unanticipated generic stimuli (auditory or visual)<br>• Unanticipated performed submaximally	1. 2 × 5 reps per limb: Jump-landings – lateral reactive jumps<br>2. 4 × 10-m acceleration to deceleration<br>3. 5 × 45° zig-zag runs (2 cuts a rep) -5-m between cuts<br>4. 10 × reactive y-agility (45° cut) – 5-m entry and exit*	100%	215	14 and 20	1. 2 × 5-m acceleration to deceleration-<br>2. 4 × 2.5-10 m unanticipated decelerations – auditory stimuli<br>3. 2 × 15-m zig-zag runs (2 90˚ cuts) – 5-m between cuts to 5-m deceleration<br>4. 2 × 15-m zig-zag runs relay (2 90˚ cuts) – 5-m between cuts to 5-m deceleration<br>5. 8 × reactive y-agility (60° cut) – 7.5-m entry and exit*<br>6. 3 × 5-m approach to 180°, 5-m approach to 180°, to 10-m deceleration	100%	280-310	11 and 23
**Week 4**		1. 2 × 5 reps per limb: Jump-landings – lateral reactive jumps<br>2. 4 × 20-m acceleration to deceleration<br>3. 4 × 45° zig-zag runs (2 cuts a rep) -6-m between cuts<br>4. 4 × 15-m tunnel drill – side-stepping past 2 opponents every 7.5-m<br>5. 4 × unanticipated 45° cuts (auditory stimuli) – 7.5-m entry and exit<br>6. 6 × reactive y-agility (45° cut) – 5-m entry and exit*	1. 100%<br>2. 75%+<br>3. 100%<br>4. 75%+<br>5. 75%+<br>6. 100%	282	4 and 32	1. 4 × 5-15 m unanticipated decelerations – partner stimuli*<br>2. 2 × 15-m zig-zag runs (2 90˚ cuts) – 5-m between cuts to 5-m deceleration<br>3. 2 × 30-m zig-zag runs relay (4 90˚ cuts) – 5-m between cuts to 5-m deceleration<br>4. 4 × unanticipated 45° cuts (auditory stimuli) – 5-m entry and exit<br>5. 6 × reactive y-agility (60° cut) 2 cuts – 5-m entry and exit*<br>6. 4 × 15-m tunnel drill – side-stepping past 2 opponents every 7.5-m	100%	330-370	16 and 38
**Week 5**	• Unanticipated drills performed maximally<br>• Introduction of sport specific stimuli – opponent or ball	1. 2 × 15-m curvilinear sprint (s shape)<br>2. 12 × 5-m acceleration to side-step (60˚) – 5-m exit to deceleration<br>3. 4 × 15-m tunnel drill – side-stepping past 2 opponents every 7.5-m<br>4. 4 × unanticipated 45° cuts (auditory stimuli) – 7.5-m entry and exit	100%	285	10 and 32	1. 4 x 10-15-m unanticipated decelerations – partner stimuli*<br>2. 2 x 180° races – two turns at any point across 15-m distance – partner lead*<br>3. 8 x X-agility: 1 pivot and 1 cut per rep – up to 15m*<br>4. 3 x 35-m zig-zag runs races (5 x 90˚ cuts) – 5-m between cuts to 5-m deceleration – 1st cut unanticipated	100%	~405-425	15 and 35

	5. 6 × reactive y-agility (60° cut) 2 cuts – 7.5m entry and exit*							
**Week 6**	1. 12 × 5-m acceleration to side-step (60˚) – 5-m exit to deceleration<br>2. 3 × Zig-zag races – 25-m length 4 x 45° cuts per race – 1st cut unanticipated<br>3. 4 x unanticipated 45° cuts (auditory stimuli) – 7.5-m entry and exit<br>4. 10 × reactive y-agility (60° cut) cuts – 7.5m entry and exit*	100%	345	48 (COD)	1. 4 × 10-15-m unanticipated decelerations – partner stimuli*<br>2. 2 × 180° races – two turns at any point across 15-m distance – partner lead*<br>3. 8 × X-agility: 1 pivot and 1 cut per rep – up to 15m*<br>4. 3 × 35-m zig-zag runs races (5 x 90˚ cuts) – 5-m between cuts to 5-m deceleration – 1st cut unanticipated	100%	~405-425	15 and 35

Additional information:

1. 30-60 seconds' rest provided between 100% effort reps. 2 minutes' rest provide between exercises
2. All CODs and decelerations to be performed with the aim of modified braking and COD strategy
3. Feedback to be provided to each player after each rep regarding braking strategy/ COD technique

Key: * = Alternate between leading and reacting / attacking and defending; COD = Change of direction; PFC = Penultimate foot contact; Dec: Deceleration.

Table S2. Task-specific pre-post changes in CMAS deficits.

CMAS criteria	IG – Pre-to-post changes in CMAS criteria				CG – Pre-to-post changes in CMAS criteria			
	Right cut		Left cut		Right cut		Left cut	
	n of deficits	%	n of deficits	%	n of deficits	%	n of deficits	%
No clear PFC braking strategy	-5	-23.8	-12	-57.1	-8	-33.3	-2	-8
Wide foot plant	3	33.3	-2	-22.2	1	7.7	2	22
Hip internal rotation	-7	-100.0	-5	-100.0	-1	-25.0	-1	-33
Initial knee valgus	-9	-39.1	-5	-27.8	-2	-9.1	-2	-11
Internal/external foot	3	27.3	-5	-26.3	1	5.6	9	60
Upright trunk	2	22.2	3	50.0	-5	-41.7	1	9
Lateral trunk flexion	-4	-26.7	-5	-27.8	8	66.7	-1	-5
Trunk leaning back	-4	-57.1	-5	-62.5	4	133.3	5	250
Limited Knee Flexion	-1	-16.7	-5	-50.0	-2	-12.5	6	50
Excessive Knee valgus	-12	-66.7	-5	-50.0	-3	-18.8	-3	-43

Key: CMAS: Cutting movement assessment score; PFC: Penultimate foot contact; IG Intervention group; CG: Control group; n: number.

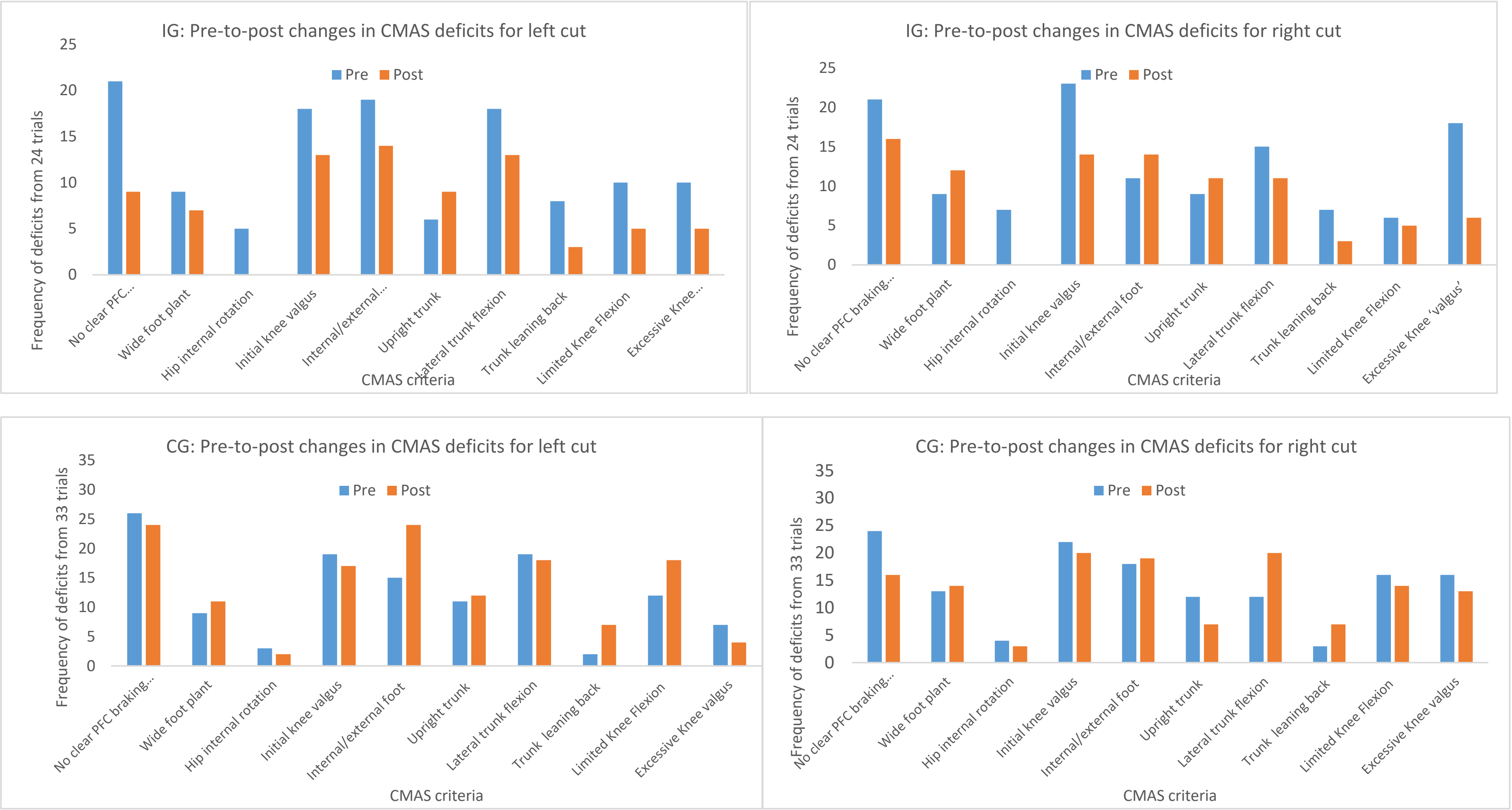

Figure S1. Cutting task-specific changes in CMAS deficits. IG: Intervention group; CG: Control group; CMAS: Cutting movement assessment score.

© 2019 by the authors. Submitted for possible open access publication under the terms and conditions of the Creative Commons Attribution (CC BY) license (http://creativecommons.org/licenses/by/4.0/).
